# Hydrophilic nanofibers in fog collectors for increased water harvesting efficiency

**DOI:** 10.1039/d0ra03939j

**Published:** 2020-06-11

**Authors:** Joanna Knapczyk-Korczak, Piotr K. Szewczyk, Daniel P. Ura, Katarzyna Berent, Urszula Stachewicz

**Affiliations:** AGH University of Science and Technology, Faculty of Metals Engineering and Industrial Computer Science, International Centre of Electron Microscopy for Materials Science al. A. Mickiewicza 30 30-059 Kraków Poland ustachew@agh.edu.pl +48 12 617 52 30; Academic Centre for Materials and Nanotechnology, AGH University of Science and Technology Poland

## Abstract

The water crisis is a big social problem and one of the solutions are the Fog Water Collectors (FWCs) that are placed in areas, where the use of conventional methods to collect water is impossible or inadequate. The most common fog collecting medium in FWC is Raschel mesh, which in our study is modified with electrospun polyamide 6 (PA6) nanofibers. The hydrophilic PA6 nanofibers were directly deposited on Raschel meshes to create the hierarchical structure that increases the effective surface area which enhances the ability to catch water droplets from fog. The meshes and the wetting behavior were investigated using a scanning electron microscope (SEM) and environmental SEM (ESEM). We performed the fog water collection experiments on various configurations of Raschel meshes with hydrophilic PA6 nanofibers. The addition of hydrophilic nanofibers allowed us to obtain 3 times higher water collection rate of collecting water from fog. Within this study, we show the innovative and straightforward way to modify the existing technology that improves water collection by changing the mechanisms of droplet formation on the mesh.

## Abstract 2

Modification of Raschel meshes used for fog water collectors with PA6 nanofibers allow to obtain 300% higher water collection rate in collecting water from fog.

## Graphical abstract

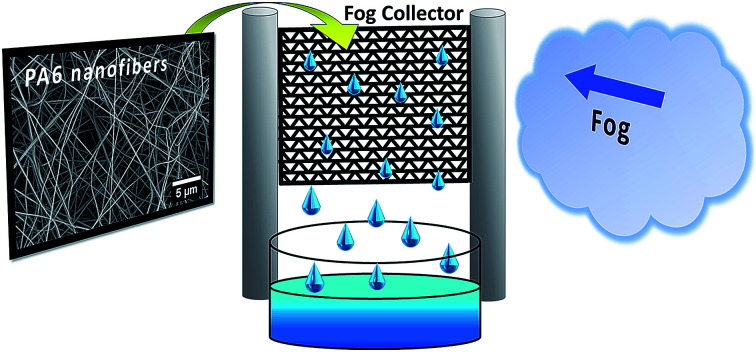

## 1. Introduction

The basis of life is water. And although the oceans cover more than 70% of the Earth's surface, the global water crisis affects at least two-thirds of the human population living in areas that lack water.^1^ Moreover, the problem worsens each year as global climate change takes place.^2^ While collecting water from fog may sound revolutionary, it is a simple technique, which can be observed in nature.^3^ For millions of years, nature has developed special mechanisms that allow organisms to harvest water from humid air, dew and fog. The best examples are Namib desert beetles (*Stenocara gracilipes* and *Onymacris unguicularis*)^4^ or cactus (*Opuntia microdasys*),^5^ which have created a hydrophobic–hydrophilic system for the water collection.^6^ The Namib desert beetle is bioinspiration for surface functionalization to demonstrate hydrophobicity.^7–9^ A lot of reptiles live in the desert regions such as a thorny devil lizard (*Moloch horridus*), which collects water from the air by its skin.^10^ Their body created the specific mechanism with many micro-channel, which allow them to harvest the moisture from the environment. The example of natural fog collector is a spider web that combines the hydrophobic and hydrophilic properties, creating the natural Janus fibers system.^11^ Among many biomimetic strategies, Pinchasik *et al.*^12^ indicated the three main aspects that are important in fog water harvesting such as condensation, adhesion and guided transport of water droplets. Water easily condenses on hydrophilic surfaces in opposition to hydrophobic surfaces with the minimum pinning of the water droplet. In the case of biphilic surfaces, the border between the hydrophobic and hydrophilic parts is the origin of water pinning observed in nature.^6^ Indeed the wettability gradient on surfaces drives droplets motion necessary for water transportation in collecting systems. We can mimic the mechanisms that were developed by nature and apply those strategies in material and structure design to create systems collecting water from fog with extraordinary efficiency.^13,14^ One of the most popular solutions are Fog Water Collectors (FWCs),^15,16^ that use meshes with specialized weave stretched on the special stand.^17–19^ FWCs collect water from droplets ranging from 1–30 μm, which collide with the fibers of the mesh or other medium.^20^ Many factors have significant influence to the efficiency of FWCs like: fog velocity, the diameter of the fog droplets and liquid water content in fog.^17,20^ Real important are wetting and aerodynamic characteristics of the collecting medium and the relation between the diameter or width of the mesh fibers or ribbons.^21–23^ The fog passes through the mesh can be collect by the collecting medium such as fibers, ribbons or wires.^22^ Importantly, the efficiency of water collection strongly depends from the fog velocity^21^ and the porosity of the mesh.^24^

Therefore, we incorporated the hydrophilic polyamide 6 (PA6) nanofibers in the existing Raschel mesh system,^25,26^ to increase the water collection efficiency. For this purpose, we use electrospinning to deposit nanofibers directly on the Raschel mesh. Electrospinning works by applying the electric field to the solution, which causes it to form a polymer jet.^27–29^ While the polymer jet is exposed to the environment, the solvent rapidly evaporates, which leads to deposition of polymer on the desired surface in form of fibers.^30–32^ The randomly electrospun fibers form membranes with porosity above 90%.^33^ The electrospun fibers have a wide range of applications in such as filtration,^34–37^ structured composites,^38–40^ water^41,42^ and energy harvesting,^36,43–45^ as well as medical applications, especially tissue engineering.^46–48^ Our focus on water harvesting using large surface area created by nanofibers gives the advantage of catching small water droplets. We have selected PA6 due to its mechanical properties of individual fibers,^49^ membranes. In addition, they provide a very good reinforcement in the composite structures^40,50,51^ and are hydrophilic.^52,53^ In this study we investigated the water collection rate in collecting water from fog on the Raschel meshes modified with electrospun PA6 nanofibers. We focused on extreme condition, where the fog flow velocity is very low, below 1 m s^−1^. The inspiration came from nature, where animals and plants harvest water from fog and humid air in the windless environmental conditions. Our system gives the possibility to increase the amount of collected water by our meshes in comparison to commercial mesh, which need the wind to collect water effectively.

## 2. Material and methods

### 2.1 Materials and electrospinning

**Fig. 1:**
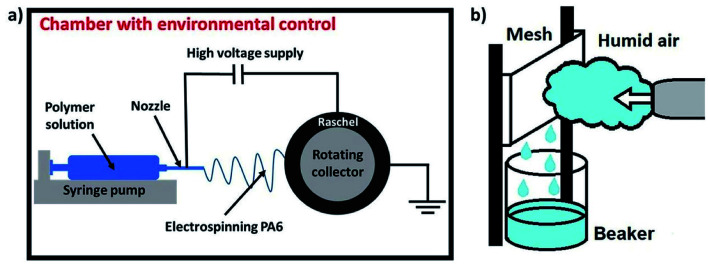
The schematics of the experimental setups for (a) electrospinning; (b) fog collection.

The polyamide 6 (PA6; BASF, Germany; *M*_w_ = 24 000 g mol^−1^), dried to constant weight at a temperature of 40 °C for 3 h, was dissolved in a mixture of formic and acetic acids with ratio 1 : 1_(vol.)_ (99.5%, Avantor, Poland). The PA6 solution with a concentration of 12 wt% was stirred for 4 h at an ambient temperature of 25 °C and at a constant speed of 500 rpm (IKA RCT basic, Germany). The electrospun fibers were obtained by electrospinning technique which is presented schematically in Fig. 1a. The chamber with environmental climate control (IME Technologies, The Netherlands) provided the constant temperature of 25 °C and humidity of 40% during the manufacturing process. The fibers were electrospun with the constant voltage of 16 kV applied between the stainless needle and the grounded collector. The polymer flow rate and used distance were set to 0.1 ml h^−1^ and 15 cm, respectively. The PA6 fibers were electrospun directly on the Raschel mesh, which was placed on the slowly rotating collector (10 rpm). The potential difference had to be increased to 17 kV, because of the insulating properties of Raschel mesh. The PA6 fibers were deposited on Raschel for 30 min, while the PA6 membranes by itself were electrospun for 3 h. We produced 4 types of samples with PA6 nanofibers by itself: Raschel mesh with PA6 nanofibers (R + PA6), double Raschel with PA6 nanofibers 2×(R + PA6) and one thick layer of PA6 nanofibers produced and measured separately (PA6) and then placed between two Raschel meshes (R–PA6–R).

### 2.2 Microscopy analysis

The samples were coated with 5 nm of gold (rotary-pump sputter coater Q150RS, Quorum Technologies, Laughton, U.K.) before the analysis by scanning electron microscope (SEM, Merlin Gemini II, ZEISS, Germany). The fibers morphology was investigated using the accelerating voltage, current and working distance of 3 kV, 150 pA and 5–8 mm respectively. The average PA6 fiber diameter was measured similar way as described in the previous study.^53,54^ The wetting experiments on the Raschel mesh and its combination with PA6 fibers was carried out using environmental SEM (ESEM, Versa 3D, FEI, USA). The samples were analyzed together with the same environmental conditions such as the pressure in the chamber 100 Pa. The accelerating voltage and current were set to 5 kV and 4 nA.

### 2.3 Fog collection experiments

The fog collection setup is presented in Fig. 1b, where the conventional humidifier (Beurer GmbH, Germany) was used to produce fog. The equipment performance in the fog production is 400 ml h^−1^ and its velocity reaches up to 0.19 m s^−1^. The meshes with the area of 100 cm^2^ were placed on the specially designed stand. The outlet of a fog feeder was set at an angle of 90° and with a distance of 6 cm from the mesh. The humidity in the stream of fog was above 95%.^53^ The water captured by meshes was running down to the glass beaker, which was weighted every 30 min over a 3 h experiments. The mesh after the water collection experiment was weighted to calculated the water retained inside it. The obtained water collection rate was calculated per the mesh area and the time of 1 h. The six types of samples were measured and were described by the symbols listened below: R – Raschel mesh; 2× R – a double layer of Raschel mesh; PA6 – PA6 nanofibers mesh; R + PA6 – Raschel mesh with PA6 nanofibers; 2×(R + PA6) – a double layer of Raschel mesh with PA6 nanofibers; R–PA6–R – separately produced PA6 nanofibers placed between two Raschel layers.

## 3. Results and discussion

### 3.1 Morphology and wetting of meshes

**Fig. 2:**
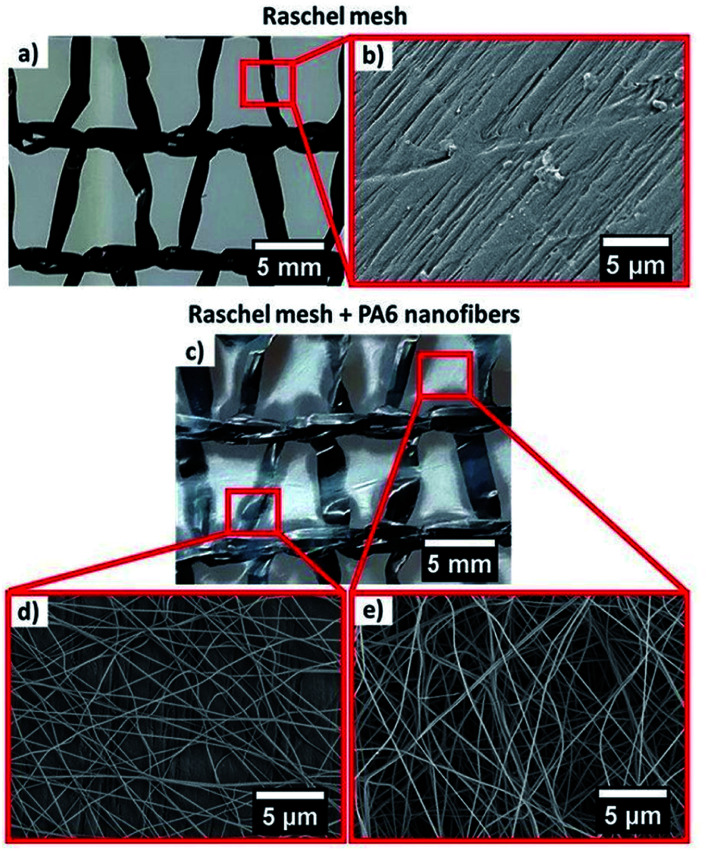
Macro- and micrographs of (a) Raschel mesh (b) close-up to the surface of the individual ribbons in the Raschel; (c) Raschel mesh with the layer of PA6 nanofibers (d) SEM of nanofibers deposited on the Raschel ribbon and (e) PA6 nanofibers network between the ribbons in the Raschel mesh.

The images and SEM micrographs of the Raschel mesh and PA6 nanofibers are shown in Fig. 2. The Raschel ribbons were characterized in a previous study and their average width reached 1.61 ± 0.12 mm and the space between ribbons in Raschel is ranging from 1.65 ± 0.22 to 3.71 ± 0.23 mm, which we managed to cover with the electrospun PA6 nanofibers. The PA6 nanofibers have the average fiber diameters of 110 ± 27 nm and 118 ± 23 nm for the PA6 deposited between ribbons and on the ribbons of the Raschel mesh, respectively. The histograms of PA6 nanofibers with their diameter distributions were reported in our previous studies.^53,54^

**Fig. 3:**
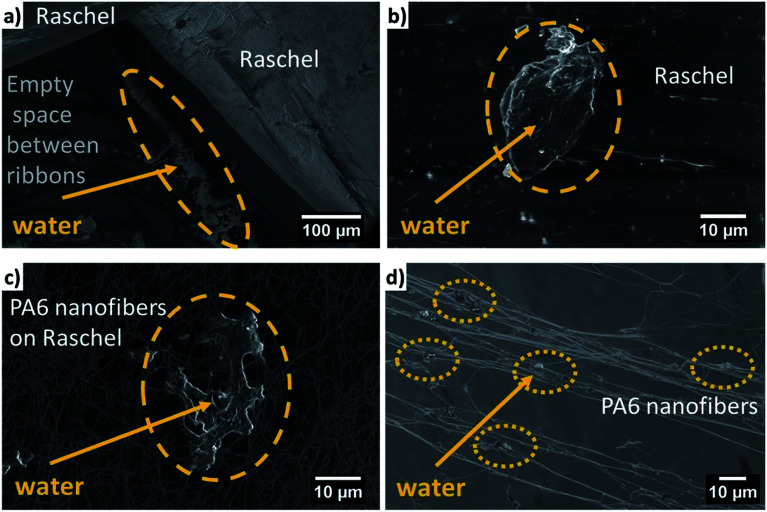
Micrographs from ESEM of wetting behavior on the samples mesh: (a) and (b) Raschel; (c) and (d) R + PA6.

The wetting of Raschel mesh and PA6 nanofibers was investigated with the ESEM, see Fig. 3. The droplets that remain on the surface of Raschel take on a characteristic oval shape, Fig. 3b. In the case of the connection of Raschel with PA6 fibers the water enters and stays between the PA6 fibers, where it freezes due to decreased pressure in the ESEM chamber. The PA6 nanofibers are able to catch small water droplets on fibers and also between pores due to its hydrophilicity as showed in Fig. 3c and d. Additionally, the investigation with ESEM showed the hydrophobic character of Raschel mesh and hydrophilic of PA6 nanofibers, where droplets are spread between fibers and also being collected on the fibers, see Fig. 3. The addition of PA6 nanofibers increases the number of droplets collected on the mesh due to increased surface area. Also, the water collection process is accelerated by the hydrophilic nanofibers.^55,56^ As imaged with ESEM, Fig. 3, some water droplets were accumulated between ribbons in Raschel mesh.

### 3.2 Fog collection experiments

**Fig. 4:**
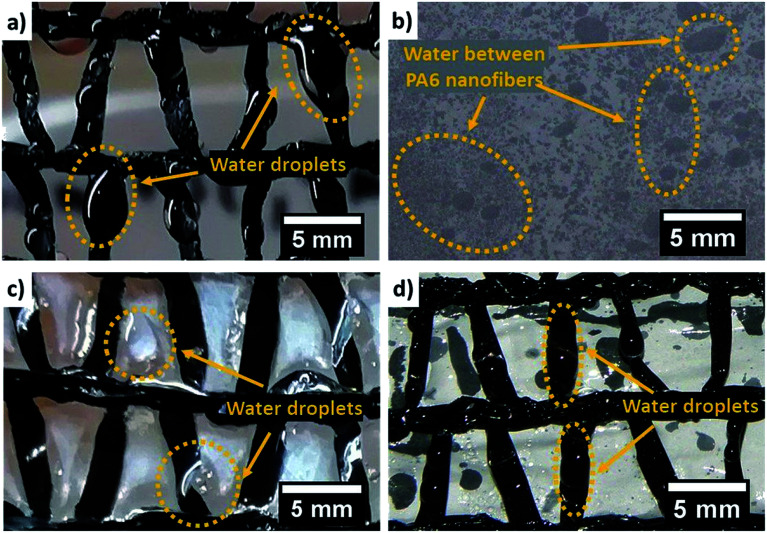
The water droplets on the meshes after 60 min exposure to fog flow from the humidifier: (a) only Raschel; (b) PA6 nanofibers; (c) Raschel with deposited on top PA6 nanofibers (R + PA6); (d) two Rachel meshes with PA6 nanofibers in between (R–PA6–R).

The shape of water droplets on the Raschel meshes is shown in Fig. 4a. The droplets deposited on the ribbons run down under their own weight and gravity to the beaker below. Often the water is trapped between the ribbons and limits the flow of fog throughout the mesh, what reduced its efficacy in collecting water.^24^ The mechanism of water harvesting on the PA6 mesh is quite different from the processes taking place on Raschel. The geometry of PA6 nanofibers provides an unique mechanism to drainage the water from the mesh in another way than the gravity.^53^ The ultra-small size of PA6 fibers allow to faster water removal from the mesh. The nanofiber meshes are characterized, with very high porosity reaching 96%, with the typical distance between fibers 1.7 μm,^40^ what allows the free flow of fog. Fig. 4b shows that the water is collected between nanofibers as they are hydrophilic, however the water is spread and we do not observe any large droplets sticking out of the mesh.

This hydrophilic behavior of PA6 nanofiber meshes was also confirmed with ESEM observations indicated in Fig. 3c. Therefore, we combine the already commercially used Raschel mesh with PA6 nanofibers to add the hydrophilic part to increase their water collection rate, as shown in Fig. 4c and d. The water droplets condense on both ribbons and nanofibers. Importantly, the PA6 nanofibers increase significantly the effective area of catching the droplets. From the other hand the water saturated between nanofiber may reduce the wind passage reducing collected water. This effect is strongly depended on the wind speed as the high winds reaching even the speed from 10 to 70 m s^−1^, may be able to shake the captured water between nanofibers. In this study we performed experiments in so called low wind conditions, with the fog flow velocity of 0.19 m s^−1^.^53^ The meshes with the larger fiber diameter, approximately 5 μm, are characterized with the increased space between fibers, as we observed in case of electrospun PS fibers, however the collected water from fog was lower in the laboratory conditions.^53^

**Fig. 5:**
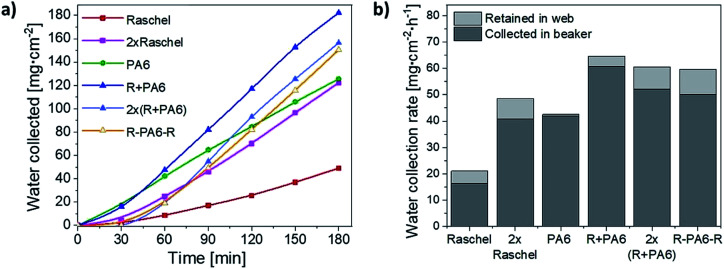
The water collected: (a) during the process per every 30 min; (b) calculated per 1 hour for Raschel mesh, double Raschel mesh (2× Raschel), PA6 nanofibers, Raschel with PA6 nanofibers (R + PA6), double Raschel with PA6 nanofibers 2×(R + PA6) and one layer of PA6 nanofibers between two Raschel meshes (R–PA6–R).

In Fig. 5, we compared the water collection rate between commercial Raschel and its combination with our PA6 nanofibers. We notice that the best water collection is for one layer of Raschel with PA6 nanofibers (R + PA6), where the ribbons in the Raschel mesh are also covered with the nanofibers as showed Fig. 2d. The R + PA6 mesh better drained water into the beaker compared to the double Raschel meshes (2× Raschel), 2 layers of Raschel with PA6 nanofibers (2×(R + PA6)) and one layer of separately produced PA6 nanofibers placed between double Raschel (R–PA6–R), see Fig. 5. These results confirm, that increase of the water collecting area is very important, however, the extra layers reduce possibility to pass the fog through the fog collecting system. In case of separately produced PA6 nanofibers, the membrane was deposited for longer to obtain easy to handle sample with higher thickness, which was controlled by the time of electrospinning. The samples R + PA6 mesh, where the PA6 nanofibers were directly electrospun on the Raschel meshes were easier to handle and perform the water harvesting experiments. Importantly, the addition of hydrophilic PA6 nanofibers increased the water collection rate by 3 times in comparison to Raschel mesh. The mesh from double Raschel allows to obtain a similar result for water collected in beaker like in the case of PA6 mesh. However, the geometry of Raschel obstructed the drainage system for water, what decreases water collection rate by 29%, see Fig. 5.

In the previous study, we investigated the wetting behavior on the hydrophobic PS microfibers and hydrophilic PA6 nanofibers, which allow to obtain the hierarchical composites to water collection.^53^ We confirmed that hydrophilic material retained accumulated water for longer, what gives the water more time to drain into the beaker. As indicated by Park *et al.*,^24^ water retained in the mesh decreases the collection efficiency in fog harvesting in the atmospheric conditions. In case of the laboratory experiments, the fog velocity is low and often the droplet size distribution of the nebulizer used is smaller than the size distribution of atmospheric fogs observed in fog harvesting. However, electrospun fibers able to improve water drainage, and increasing collection rates. These solutions are often found in nature, which adapts organisms to live in an extremely hostile environment without freshwater reservoirs.^3^ The key element in proposed modification of the commercially used Raschel meshes are PA6 nanofibers that have been proved very stable mechanically as individual fibers^49^ and in meshes,^53,57^ for composite constructions^40^ and also in the cryo environment.^33^ Our previous study confirmed the good mechanical properties of randomly oriented PA6 nanofiber meshes, where the average maximum stress reached 1.24 MPa.^53^ Importantly, the deposited PA6 nanofibers on the Raschel mesh modify the surface properties of ribbons by increasing its roughness and the hierarchical elements to mesh as their sizes are in the range of 100 nm. Azad *et al.*^58^ provided evidence of how important is the surface structure of the system used to harvest water effectively. The best results showed the structure based on the fibers with round, oval and rectangular shapes in their cross sections.

Particular importance in the fog harvesting has the combination of hydrophobic and hydrophilic surface properties. Lee *et al.*^59^ proposed the construction inspired by the cactus stem, which based on the superhydrophilic–superhydrophobic system. Their cylindrical double structural system reached of 209 mg cm^−2^ h^−1^ of water recovery. Also Cao *et al.*^60^ proposed a hydrophobic–hydrophilic Janus System based on the hydrophobic copper mesh and hydrophilic cotton absorbent. This system with the area of 2 × 2 cm^2^ reached of 0.31 ± 0.03 g of water per piece. The performance of water harvesting from fog depends on environmental conditions like fog flow velocity and ambient humidity and also from the FWC construction. In both cases the fog flow velocity was 3.5 times faster from our system and reached ≈0.7 m s^−1^. Our model of Raschel with PA6 nanofibers has lower water collection rate due to differences in the experimental conditions, however, it shows the desired ability to collect water in regions with the low fog flow velocity. The water collection rate reaching 64 mg cm^−2^ h^−1^ is a great achievement in such laboratory conditions. Additionally, we show a possibility to modify Raschel meshes at a low cost by stable electrospinning of PA6 nanofibers. The technology for production of high amounts of electrospun nanofibers is already present in the field of air filtration.^61^

## 4. Conclusion

In conclusion the water collection rate of single layer of commercial Raschel mesh can be increased by 300% in a very simple way by incorporation hydrophilic PA6 nanofibers layer in a single step manufacturing method. It is possible thanks to increasing the water collection area and improving the water drainage mechanism. This solution allows to collect water in more effective way in the windless or low wind speed conditions. This solution shows the new approach and future development path of creating more efficient FWC constructions. In terms of water harvesting, we need to keep in mind that water harvesting innovations must be coupled with better water management. People need to find new ways of storing or capturing water in places that are becoming scarcer with this vital resource. The water crisis is a big social problem and it requires the material scientists working together with applied environmental units to be able to bring innovation to the fog collectors.

## Data availability

The data supporting this article are found within the text. Any additional data and the data that support the plots within this paper are available from the corresponding author upon reasonable request.

## Conflicts of interest

The authors declare that they have no known competing financial interests or personal relationships that could have appeared to influence the work reported in this paper.

## Supplementary Material

## References

[cit1] Willis K. , The Sustainable Development Goals Report 2019, 2019

[cit2] WWAP (UNESCO World Water Assessment Programme) , The United Nations World Water Development Report 2019, Leaving No One Behind, 2019

[cit3] Joel A.-C. , Buchberger G. and Comanns P., in Functional Surfaces in Biology III, ed. S. N. Gorb and E. V. Gorb, Springer International Publishing, Cham, 2017, vol. 10, pp. 93–106

[cit4] Bhushan B. (2019). Bioinspired water collection methods to supplement water supply. Philos. Trans. R. Soc., A.

[cit5] Gurera D., Bhushan B. (2020). Designing bioinspired conical surfaces for water collection from condensation. J. Colloid Interface Sci..

[cit6] Bhushan B. (2019). Lessons from nature for green science and technology: an overview and bioinspired superliquiphobic/philic surfaces. Philos. Trans. R. Soc., A.

[cit7] Oyola-Reynoso S., Tevis I. D., Chen J., Chang B. S., Çinar S., Bloch J. F., Thuo M. M. (2016). Recruiting physisorbed water in surface polymerization for bio-inspired materials of tunable hydrophobicity. J. Mater. Chem. A.

[cit8] Brown P. S., Bhushan B. (2016). Bioinspired materials for water supply and management: water collection, water purification and separation of water from oil. Philos. Trans. R. Soc., A.

[cit9] Bhushan B. (2020). Design of water harvesting towers and projections for water collection from fog and condensation. Philos. Trans. R. Soc., A.

[cit10] Comanns P., Esser F. J., Kappel P. H., Baumgartner W., Shaw J., Withers P. C. (2017). Adsorption and movement of water by skin of the Australian thorny devil (Agamidae: Moloch horridus). R. Soc. Open Sci..

[cit11] Szewczyk P. K., Knapczyk-Korczak J., Ura D. P., Metwally S., Gruszczyński A., Stachewicz U. (2018). Biomimicking wetting properties of spider web from Linothele megatheloides with electrospun fibers. Mater. Lett..

[cit12] Pinchasik B.-E., Kappl M., Butt H. J. (2016). Small Structures, Big Droplets: The Role of Nanoscience in Fog Harvesting. ACS Nano.

[cit13] Bai H., Zhang C., Long Z., Geng H., Ba T., Fan Y., Yu C., Li K., Cao M., Jiang L. (2018). A hierarchical hydrophilic/hydrophobic cooperative fog collector possessing self-pumped droplet delivering ability. J. Mater. Chem. A.

[cit14] Zhou H., Zhang M., Li C., Gao C., Zheng Y. (2018). Excellent Fog-Droplets Collector via Integrative Janus Membrane and Conical Spine with Micro/Nanostructures. Small.

[cit15] Schemenauer R. S., Cereceda P. (1994). A Proposed Standard Fog Collector for Use in High-Elevation Regions. J. Appl. Meteorol..

[cit16] Schemenauer R. S., Cereceda P. (1994). The Role of Wind in Rainwater Catchment and Fog Collection. Water Int..

[cit17] Fernandez D. M., Torregrosa A., Weiss-Penzias P. S., Zhang B. J., Sorensen D., Cohen R. E., McKinley G. H., Kleingartner J., Oliphant A., Bowman M. (2018). Fog Water Collection Effectiveness: Mesh Intercomparisons. Aerosol Air Qual. Res..

[cit18] Rajaram M., Heng X., Oza M., Luo C. (2016). Enhancement of fog-collection efficiency of a Raschel mesh using surface coatings and local geometric changes. Colloids Surf., A.

[cit19] Rivera J. de D., Lopez-Garcia D. (2015). Mechanical characteristics of Raschel mesh and their application to the design of large fog collectors. Atmos. Res..

[cit20] Schemenauer R. S., Cereceda P. (1991). Fog-Water Collection in Arid Coastal Locations. Ambio.

[cit21] Rivera J. de D. (2011). Aerodynamic collection efficiency of fog water collectors. Atmos. Res..

[cit22] Schunk C., Trautwein P., Hruschka H., Frost E., Dodson L., Derhem A., Bargach J., Menzel A. (2018). Testing water yield, efficiency of different meshes and water quality with a novel fog collector for high wind speeds. Aerosol Air Qual. Res..

[cit23] Azeem M., Guérin A., Dumais T., Caminos L., Goldstein R. E., Pesci A. I., De Dios Rivera J., Torres M. J., Wiener J., Campos J. L., Dumais J. (2020). Optimal Design of Multilayer Fog Collectors. ACS Appl. Mater. Interfaces.

[cit24] Park K. C., Chhatre S. S., Srinivasan S., Cohen R. E., McKinley G. H. (2013). Optimal design of permeable fiber network structures for fog harvesting. Langmuir.

[cit25] Holmes R., Rivera J. de D., de la Jara E. (2015). Large fog collectors: new strategies for collection efficiency and structural response to wind pressure. Atmos. Res..

[cit26] Klemm O., Schemenauer R. S., Lummerich A., Cereceda P., Marzol V., Corell D., Van Heerden J., Reinhard D., Gherezghiher T., Olivier J., Osses P., Sarsour J., Frost E., Estrela M. J., Valiente J. A., Fessehaye G. M. (2012). Fog as a fresh-water resource: overview and perspectives. Ambio.

[cit27] Reneker D. H., Chun I. (1996). Nanometre diameter fibres of polymer, produced by electrospinning. Nanotechnology.

[cit28] Theron S. A., Zussman E., Yarin A. L. (2004). Experimental investigation of the governing parameters in the electrospinning of polymer solutions. Polymer.

[cit29] Shin Y. M., Hohman M. M., Brenner M. P., Rutledge G. C. (2001). Experimental characterization of electrospinning: the electrically forced jet and instabilities. Polymer.

[cit30] Xue J., Wu T., Dai Y., Xia Y. (2019). Electrospinning and electrospun nanofibers: methods, materials, and applications. Chem. Rev..

[cit31] Ma M., Hill R. M., Rutledge G. C. (2008). A review of recent results on superhydrophobic materials based on micro- and nanofibers. J. Adhes. Sci. Technol..

[cit32] Arinstein A., Zussman E. (2011). Electrospun polymer nanofibers: mechanical and thermodynamic perspectives. J. Polym. Sci., Part B: Polym. Phys..

[cit33] Stachewicz U., Bailey R. J., Zhang H., Stone C. A., Willis C. R., Barber A. H. (2015). Wetting Hierarchy in Oleophobic 3D Electrospun Nanofiber Networks. ACS Appl. Mater. Interfaces.

[cit34] Qin X. and Subianto S., in Electrospun Nanofibers, ed. M. Afshari, Woodhead Publishing, 2017, pp. 449–466

[cit35] Rahman M. M., Thakkar A. (2016). Use of Nano Fibers in Filtration-A Review. Int. J. Sci. Res. Dev..

[cit36] Alam M. M., Ghosh S. K., Sultana A., Mandal D. (2018). An Effective Wind Energy Harvester of Paper Ash-Mediated Rapidly Synthesized ZnO Nanoparticle-Interfaced Electrospun PVDF Fiber. ACS Sustainable Chem. Eng..

[cit37] Wang J., Zhao W., Wang B., Pei G., Li C. (2017). Multilevel-layer-structured polyamide 6/poly(trimethylene terephthalate) nanofibrous membranes for low-pressure air filtration. J. Appl. Polym. Sci..

[cit38] Gernhardt M., Peng L., Burgard M., Jiang S., Förster B., Schmalz H., Agarwal S. (2018). Tailoring the Morphology of Responsive Bioinspired Bicomponent Fibers. Macromol. Mater. Eng..

[cit39] Peng L., Jiang S., Seuß M., Fery A., Lang G., Scheibel T., Agarwal S. (2016). Two-in-One Composite Fibers with Side-by-Side Arrangement of Silk Fibroin and Poly(l -lactide) by Electrospinning. Macromol. Mater. Eng..

[cit40] Stachewicz U., Modaresifar F., Bailey R. J., Peijs T., Barber A. H. (2012). Manufacture of void-free electrospun polymer nanofiber composites with optimized mechanical properties. ACS Appl. Mater. Interfaces.

[cit41] Dong H., Wang N., Wang L., Bai H., Wu J., Zheng Y., Zhao Y., Jiang L. (2012). Bioinspired electrospun knotted microfibers for fog harvesting. ChemPhysChem.

[cit42] Ganesh V. A., Ranganath A. S., Baji A., Raut H. K., Sahay R., Ramakrishna S. (2017). Hierarchical Structured Electrospun Nanofibers for Improved Fog Harvesting Applications. Macromol. Mater. Eng..

[cit43] Agarwal S., Greiner A., Wendorff J. H. (2013). Functional materials by electrospinning of polymers. Prog. Polym. Sci..

[cit44] Szewczyk P. K., Gradys A., Kim S. K., Persano L., Marzec M., Kryshtal A., Busolo T., Toncelli A., Pisignano D., Bernasik A., Kar-Narayan S., Sajkiewicz P., Stachewicz U. (2020). Enhanced Piezoelectricity of Electrospun Polyvinylidene Fluoride Fibers for Energy Harvesting. ACS Appl. Mater. Interfaces.

[cit45] Busolo T., Ura D. P., Kim S. K., Marzec M. M., Bernasik A., Stachewicz U., Kar-Narayan S. (2019). Surface potential tailoring of PMMA fibers by electrospinning for enhanced triboelectric performance. Nano Energy.

[cit46] Hong J., Yeo M., Yang G. H., Kim G. (2019). Cell-Electrospinning and Its Application for Tissue Engineering. Int. J. Mol. Sci..

[cit47] Metwally S., Karbowniczek J. E., Szewczyk P. K., Marzec M. M., Gruszczyński A., Bernasik A., Stachewicz U. (2019). Single-Step Approach to Tailor Surface Chemistry and Potential on Electrospun PCL Fibers for Tissue Engineering Application. Adv. Mater. Interfaces.

[cit48] Flaig F., Ragot H., Simon A., Revet G., Kitsara M., Kitasato L., Hébraud A., Agbulut O., Schlatter G. (2020). Design of Functional Electrospun Scaffolds Based on Poly(glycerol sebacate) Elastomer and Poly(lactic acid) for Cardiac Tissue Engineering. ACS Biomater. Sci. Eng..

[cit49] Hang F., Lu D., Bailey R. J., Jimenez-Palomar I., Stachewicz U., Cortes-Ballesteros B., Davies M., Zech M., Bödefeld C., Barber A. H. (2011). *In situ* tensile testing of nanofibers by combining atomic force microscopy and scanning electron microscopy. Nanotechnology.

[cit50] Jiang S., Duan G., Hou H., Greiner A., Agarwal S. (2012). Novel layer-by-layer procedure for making nylon-6 nanofiber reinforced high strength, tough, and transparent thermoplastic polyurethane composites. ACS Appl. Mater. Interfaces.

[cit51] Stachewicz U., Hang F., Barber A. H. (2014). Adhesion anisotropy between contacting electrospun fibers. Langmuir.

[cit52] Stachewicz U., Barber A. H. (2011). Enhanced wetting behavior at electrospun polyamide nanofiber surfaces. Langmuir.

[cit53] Knapczyk-Korczak J., Ura D. P., Gajek M., Marzec M. M., Berent K., Bernasik A., Chiverton J. P., Stachewicz U. (2020). Fiber-Based Composite Meshes with Controlled Mechanical and Wetting Properties for Water Harvesting. ACS Appl. Mater. Interfaces.

[cit54] Szewczyk P. K., Ura D. P., Metwally S., Knapczyk-Korczak J., Gajek M., Marzec M. M., Bernasik A., Stachewicz U. (2019). Roughness and Fiber Fraction Dominated Wetting of Electrospun Fiber-Based Porous Meshes. Polymers.

[cit55] Seo D., Lee J., Lee C., Nam Y. (2016). The effects of surface wettability on the fog and dew moisture harvesting performance on tubular surfaces. Sci. Rep..

[cit56] Choo S., Choi H.-J., Lee H. (2015). Water-collecting behavior of nanostructured surfaces with special wettability. Appl. Surf. Sci..

[cit57] Stachewicz U., Peker I., Tu W., Barber A. H. (2011). Stress delocalization in crack tolerant electrospun nanofiber networks. ACS Appl. Mater. Interfaces.

[cit58] Azad M. A. K., Krause T., Danter L., Baars A., Koch K., Barthlott W. (2017). Fog Collection on Polyethylene Terephthalate (PET) Fibers: Influence of Cross Section and Surface Structure. Langmuir.

[cit59] Lee S. J., Ha N., Kim H. (2019). Superhydrophilic–Superhydrophobic Water Harvester Inspired by Wetting Property of Cactus Stem. ACS Sustainable Chem. Eng..

[cit60] Cao M., Xiao J., Yu C., Li K., Jiang L. (2015). Hydrophobic/Hydrophilic Cooperative Janus System for Enhancement of Fog Collection. Small.

[cit61] Lv D., Zhu M., Jiang Z., Jiang S., Zhang Q., Xiong R., Huang C. (2018). Green Electrospun Nanofibers and Their Application in Air Filtration. Macromol. Mater. Eng..

